# Visualizing Micro-anatomical Structures of the Posterior Cornea with Micro-optical Coherence Tomography

**DOI:** 10.1038/s41598-017-11380-0

**Published:** 2017-09-07

**Authors:** Si Chen, Xinyu Liu, Nanshuo Wang, Xianghong Wang, Qiaozhou Xiong, En Bo, Xiaojun Yu, Shufen Chen, Linbo Liu

**Affiliations:** 1 School of Electrical and Electronic Engineering, Nanyang Technological University, Singapore, 639798 Singapore; 2 School of Chemical and Biomedical Engineering, Nanyang Technological University, Singapore, 637459 Singapore

## Abstract

Diagnosis of corneal disease and challenges in corneal transplantation require comprehensive understanding of corneal anatomy, particularly that of the posterior cornea. Micro-optical coherence tomography (µOCT) is a potentially suitable tool to meet this need, owing to its ultrahigh isotropic spatial resolution, high image acquisition rate and depth priority scanning mode. In this study, we explored the ability of µOCT to visualize micro-anatomical structures of the posterior cornea *ex vivo* and *in vivo* using small and large animals. µOCT clearly delineated cornea layers and revealed micro-anatomical structures, including not only polygonal endothelial cells, stellate keratocytes, collagen fibres and corneal nerve fibres but also new structures such as the dome-shaped basolateral side of endothelial cells and lattice structures at the interface between endothelium and Descemet’s membrane. Based on these observations, a short post-harvest longitudinal study was conducted on rat cornea to test the feasibility of using µOCT to monitor the quality of endothelial cells. This study successfully reveals a series of morphological features and pathological changes in the posterior cornea at the cellular level *in situ* and in real time with µOCT. These findings enrich knowledge of corneal anatomy and suggest that µOCT may be a promising imaging tool in corneal transplantation.

## Introduction

Corneal homeostasis can be perturbed by a variety of pathological conditions, such as trauma, infection and nutritional, inherited and degenerative disorders, thus resulting in corneal edema or haze^1, 2^. Second only to cataract, corneal disease is a major cause of visual impairment or blindness worldwide^2, 3^. Corneal transplantation remains the most effective method for visual restoration after corneal clarity is irreversibly destroyed^2^. In developed countries, up to 50% of all corneal transplantations are performed to treat endothelial dystrophy^4, 5^. Currently, endothelial keratoplasty (EK) is widely used for the treatment of endothelial decompensation because of its rapid and predictable visual rehabilitation and low risk of complications, such as transplant rejection and the astigmatism that often occurs with penetrating keratoplasty (PK). However, donor graft for EK, particularly for Descemet’s membrane endothelial keratoplasty (DMEK), is extremely thin, thus making it difficult to prepare corneal grafts and increasing the risks of endothelial cell loss, allograft dislocation and disattachment^2^. Recent progress in understanding of corneal anatomy, owing to the use of the “Big Bubble” technique for corneal transplantation, has contributed to an innovation in EK and pre-Descemet EK (PDEK) in which the donor pre-Descemet’s layer (PDL) together with Descemet’s membrane (DM) and endothelium are transplanted with the aim of decreasing technical complexities and postoperative complications in DMEK^6, 7^. Consequently, accurate delineation of the posterior fine layers, i.e., the PDL, DM and endothelium as well as the corneal allograft interface, in real time and *in situ* would be of great significance in pre-, intra-, and post- operative evaluation of EK. Likewise, precise depiction of posterior corneal layers would also assist with deep anterior lamellar keratoplasty (DALK), which is currently hindered by the technical difficulty in separating the posterior stroma from the DM, thus resulting in intraoperative perforation at a rate up to 4–39%^2, 8^.

On the other hand, the quality of donor endothelial cells is the critical parameter that determines corneal graft survival in corneal transplantation^9^, and it remains a challenge for eye banks to optimize storage strategies to maximally preserve viable endothelial cells and other corneal components, especially in areas that face a shortage of donor corneas^10, 11^. Therefore, successful and efficient visualization of cellular and extracellular components *in situ* would provide valuable information for longitudinal assessment of eye bank corneas.

Currently, improvements in technology have made it possible to investigate corneal structures at the cellular level *in situ* and in real time. Noncontact specular microscopy (SM) is the most commonly used imaging tool in clinics for non-invasive assessment of endothelial cells; however, the information acquired is limited to the apical surface of the endothelium and even mild corneal edema may lead to blurred images^12, 13^. By contrast, *in vivo* confocal microscopy (IVCM) allows for visualization of all corneal layers at the cellular level, and image acquisition is not sensitive to slight corneal edema^12, 14^. However, patient discomfort caused by contact and anaesthetic is still a significant issue limiting the application of IVCM. Moreover, although cross-sectional images can be achieved by three-dimensional (3D) reconstruction of *en face* images, it is difficult to differentiate the thin layers of the posterior cornea with an axial resolution of 4–25 µm^14^. In addition, the high transverse resolution (<1 µm) is achieved at the expense of field of view (~400 µm × 400 µm), thus making it troublesome for repeated investigation of the same area over time^14, 15^. Full-field optical coherence tomography (FF-OCT), which allows for both transverse and axial resolution at the cellular level (~1 µm × 1 µm), has emerged as an alternative to IVCM^16–18^. However, its long image acquisition time (~1–1.5 s per *en face* frame) makes it unsuitable for *in vivo* corneal imaging^5, 17^.

Optical coherence tomography (OCT) is a powerful tool for evaluating corneal structures because its axial resolution is decoupled from its transverse resolution and is determined by the center wavelength and bandwidth of the light source^19^. However, the axial resolution of commercially available anterior segment OCT (AS-OCT) and custom-built ultrahigh resolution OCT (UHR-OCT) instruments is limited to 5–20 µm and 3–5 µm, respectively, which is still insufficient to differentiate the DM and endothelial layer^20–22^. Bizheva *et al*. have recently used an UHR-OCT with a resolution of 1 × 5 µm (axial × lateral resolution) to successfully delineate the PDL, DM and endothelial layer^23^. However, none of these OCT systems have sufficient lateral resolution to resolve cellular level microstructures from an *en face* plane. A new-generation OCT technique, termed micro-OCT (µOCT), with an isotropic spatial resolution of 1–2 µm, can detect key cellular and subcellular components associated with arteriosclerosis, pulmonary airway disease, and intracochlear defects *ex vivo* ^24–26^. More recently, we have tested its performance in imaging hexagonal corneal endothelial cells by using a rat model^5^. However, to date, the ability of µOCT to enable visualization of micro-anatomical structures of the posterior cornea has not been fully explored. In this report, we present µOCT images that capture micro-anatomical structures of the posterior cornea in mice, rats and swine *ex vivo*. Some of these structural features, to the best of our knowledge, are reported for the first time, which should contribute to understanding of the corneal anatomy associated with corneal transplantation. Furthermore, a preliminary *in vivo* study of swine corneas was performed to test the feasibility of using current µOCT technology for potential *in vivo* imaging in humans.

## Results

### Morphological characteristics of normal posterior cornea

#### µOCT cross-sectional view

**Figure 1:**
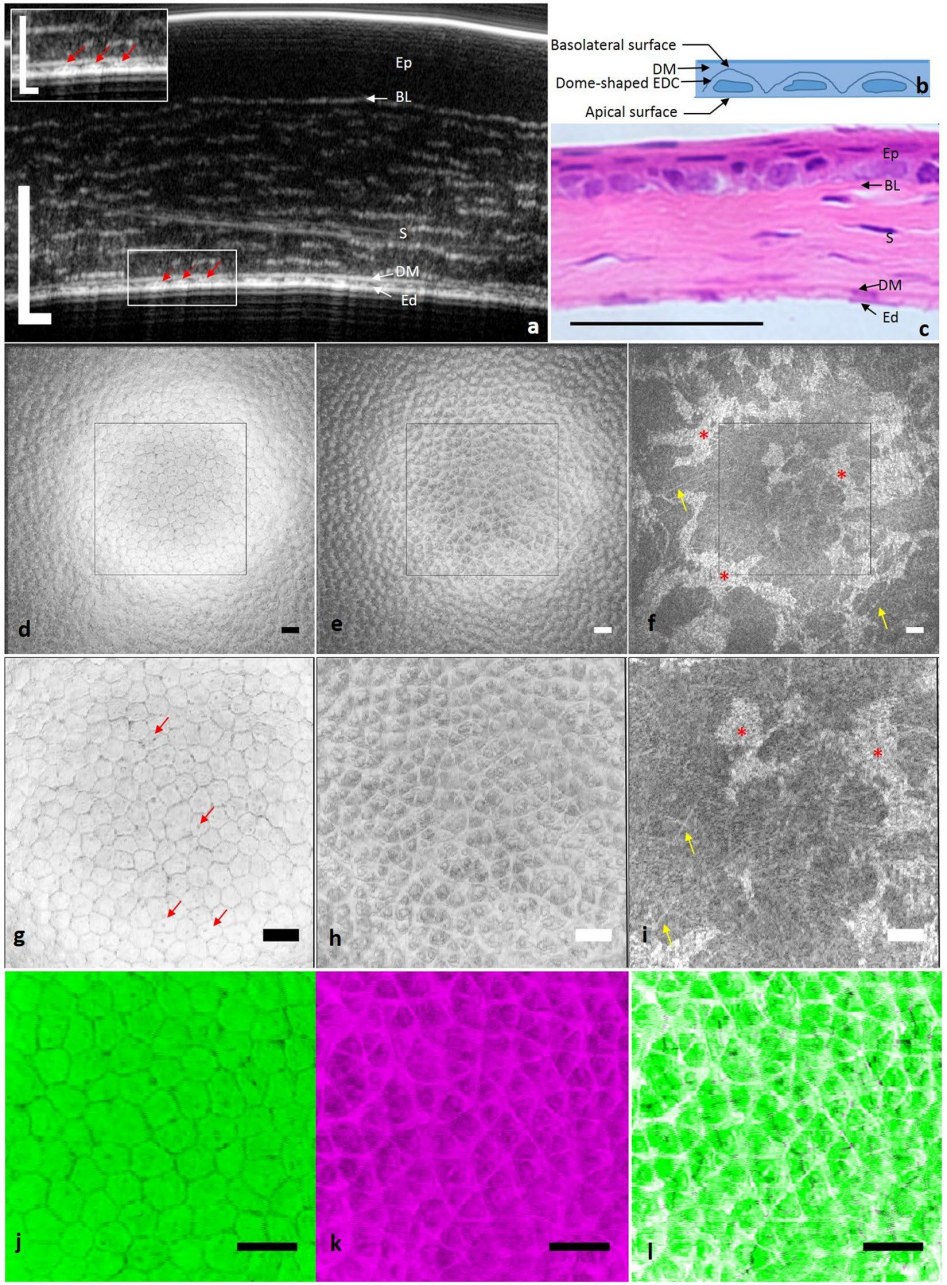
*Ex vivo* µOCT imaging of mouse cornea. (**a**) Cross-sectional µOCT image of mouse cornea. Inset is the zoomed-in view of the rectangular area; red arrows indicate endothelial cells. (**b**) Scheme of the tomographic view of endothelial cells. (**c**) Cross-sectional histological image of mouse cornea. (**d**) *En face* view of the apical side of the endothelium demonstrated regularly arranged polygonal cells with low reflective cell boundaries. (**e**) *En face* view of the interface between the endothelium and DM, corresponding to the basolateral side of the endothelium, presented a high scattering lattice. (**f**) *En face* view of posterior stroma. Stellate keratocytes (red asterisks) and linear collagen fibres (yellow arrows) were both highly reflective. (**g**–**i**) Zoomed-in view of the square region in (**d**–**f**). Dark spots are probably primary cilia of endothelial cells (red arrows in **g**). (**j**) Apical surface of endothelial cells. (**k**) Basolateral surface of endothelial cells. (**l**) Overlap of apical and basolateral surface of endothelial cells. Ep: epithelium; BL: Bowman’s layer; S: stroma; DM: Descemet’s membrane; Ed: endothelium; EDC: endothelial cell (Scale bar = 50 µm and scale bar of inset in (**a**) represents 25 µm).

**Figure 2:**
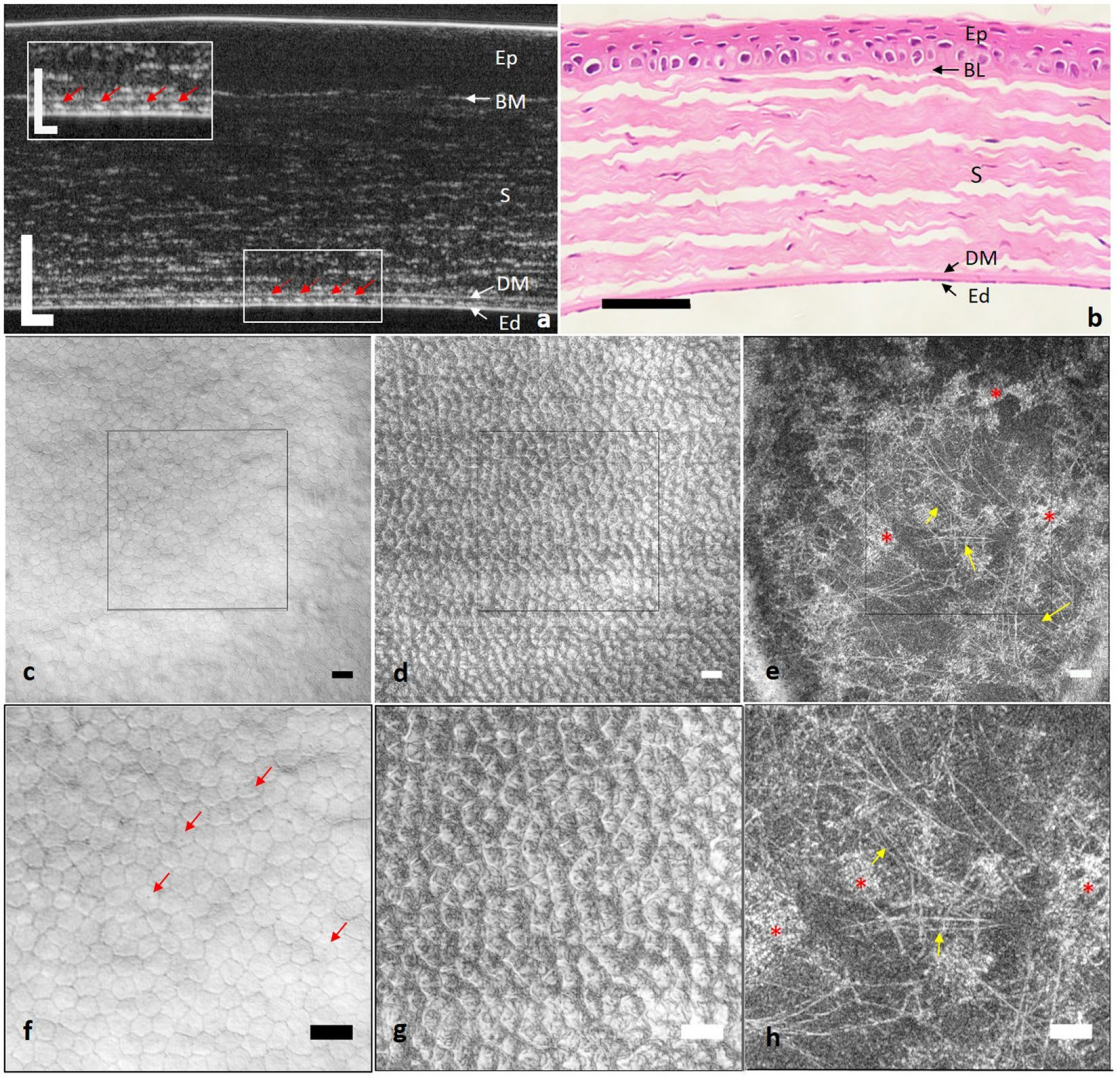
*Ex vivo* µOCT imaging of rat cornea. (**a**) Cross-sectional µOCT image of rat cornea. Inset is the zoomed-in view of the rectangular area; red arrows indicate endothelial cells. (**b**) Cross-sectional histological image of rat cornea. (**c**) *En face* view of the apical side of the endothelium demonstrated regularly arranged polygonal cells with low reflective cell boundaries. (**d**) *En face* view of the interface between the endothelium and DM, which corresponded to the basolateral side of the endothelium, presented a highly scattering lattice. (**e**) *En face* view of posterior stroma. Stellate keratocytes (red asterisks) and linear collagen fibres (yellow arrows) were both visualized. (**f**–**h**) Zoomed-in view of the square region in (**c**–**e**). Dark spots are probably cilia of endothelial cells (red arrow in **f**). Ep: epithelium; BL: Bowman’s layer; S: stroma; DM: Descemet’s membrane; Ed: endothelium (Scale bar = 50 µm and scale bar of inset in (**a**) represents 25 µm).

Representative *ex vivo* cross-sectional µOCT images of mice and rat corneas showed well-defined interfaces between adjacent posterior corneal layers (Figs 1a and 2a), as confirmed by corresponding histological images (Figs 1c and 2b). The interface between the aqueous humour and endothelium, corresponding to the apical side of endothelial cells, resulted in a continuous hyper-reflective line. The interface between the endothelium and DM, corresponding to the basolateral side of endothelial cells, appeared as an interspaced high scattering string. Each high scattering segment of the string appeared to be dome-shaped (red arrow in Figs 1a and 2a) and corresponded to one endothelial cell (Fig. 1b). DM was hypo-reflective in µOCT images with a high scattering interface with the stroma. The tomographic view of the stroma was optically heterogeneous with highly scattering linear structures running parallel to the surface of the cornea against a low reflective background. We speculated that these linear structures were collagen bundles detectable in the near infrared spectrum by µOCT.

**Figure 3:**
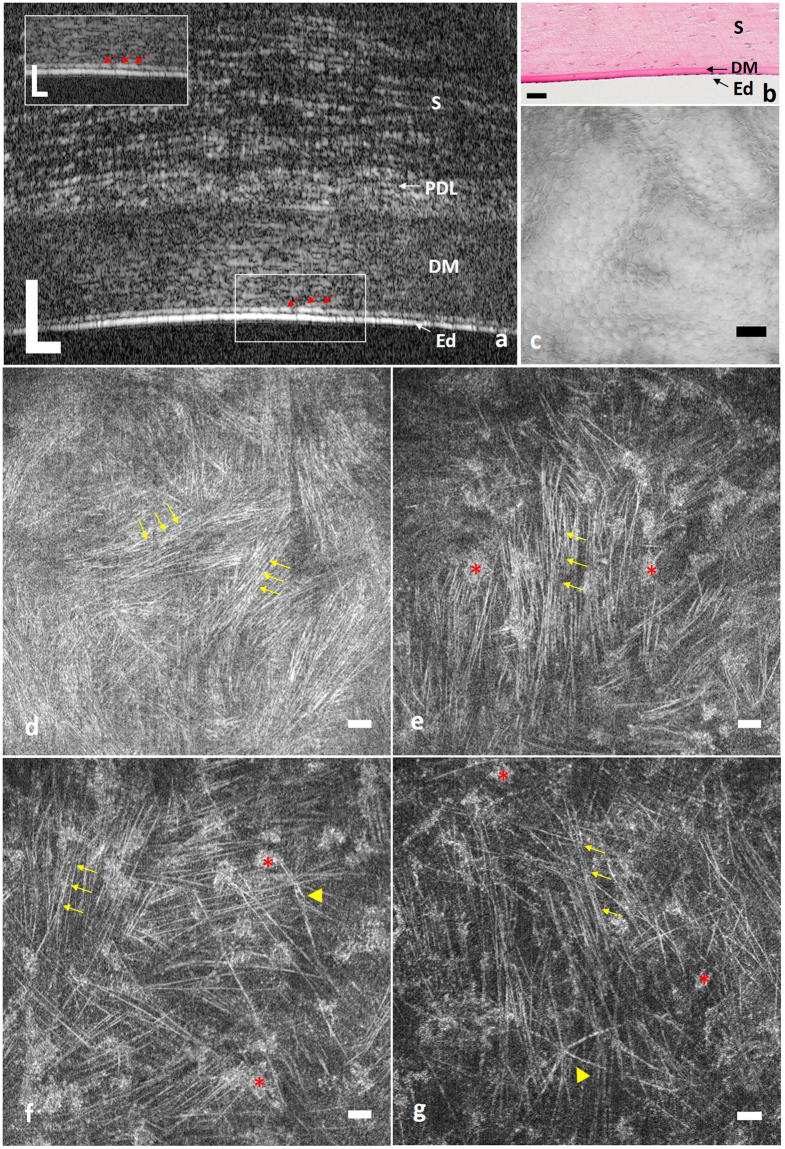
µOCT imaging of pig cornea. (**a**) *In vivo* cross-sectional µOCT image of swine posterior cornea. Inset is the zoomed-in view of the rectangular area; red arrows indicate endothelial cells. Corneal layers were clearly delineated, and collagen bundles in stroma were also visualized. (**b**) Cross-sectional histological image of posterior cornea. (**c**) *Ex vivo en face* view of the apical side of the endothelium presented regularly arranged polygonal cells. (**d**–**f**) Representative *ex vivo en face* views of posterior stroma from the lower to upper level. Both keratocytes (red asterisks) and collagen bundles (yellow arrows) were visualized, and the density of visible collagen bundles decreased from the most posterior to upper posterior stroma. Yellow triangles in (**f** and **g**) indicate nerve fibres in different patterns. S: stroma; PDL: pre-Descemet’s layer; DM: Descemet’s membrane; Ed: endothelium (Scale bar = 50 µm and scale bar of inset in (**a**) is 25 µm).

µOCT cross-sectional images of minipigs acquired *in vivo* clearly delineated the posterior corneal layers, which included the endothelium, low scattering DM and stroma (Fig. 3a). The above-mentioned observations in the *ex vivo* cross-sectional µOCT images of the posterior cornea were also visualized in images acquired *in vivo*, including the dome-shaped basolateral surface of endothelial cells (red arrow in Fig. 3a). Interestingly, we also observed a thin high scattering layer just anterior to the DM in µOCT images captured *in vivo*, which was reminiscent of the pre-DM layer (Fig. 3a).

In addition, we also quantified the endothelial thickness and DM thickness in this study. The average thickness of endothelium was measured to be 5.31 ± 0.58 µm (mouse), 4.78 ± 0.43 µm (rat) and 4.78 ± 0.70 µm (swine); while the average DM thickness was 1.96 ± 0.59 µm, 3.23 ± 0.46 µm and 32.42 ± 1.11 µm for mouse, rat and swine respectively.

#### µOCT *en face* view

Continuous *en face* views of the posterior cornea from 3D reconstruction of µOCT data are presented in Figs 1d–i, 2c,h and 3c–g. Similar morphological features were shared among mice, rats and swine. Polygonal or hexagonal endothelial cells were clearly delineated with distinct low reflective cell boundaries (Figs 1d,g, 2c,f and 3c). Endothelial cell density was 952, 1108 and 2139 per mm^2^ for mouse, rat and swine respectively. The dark spots located at the apical surfaces of endothelial cells were believed to be the root of primary cilia rather than cell nuclei, as previously speculated (red arrows in Figs 1g and 2f)^27^. This hypothesis was supported first by the observation that they were too small for nuclei^5, 27^ and second by the observation that the dark spots were found at only the apical side of the cell membrane, not at both the apical and basolateral sides. Interestingly, the *en face* images of the basolateral surface of endothelial cells demonstrated a hyper-reflective lattice, particularly in mice and rats, a finding that, to the best of our knowledge, has never been reported on the basis of confocal microscopy or FF-OCT data (Figs 1e,h, 2d and g). Orthogonal projections of this hyper-reflective lattice showed that each junction point of the lattice corresponded to one dome-shaped endothelial cell. This observation was confirmed when we overlaid the image of the endothelial apical surface with the lattice: a one-to-one match was observed between each intersection point and endothelial cell centre on the overlapped image (Fig. 1j–l). No identifiable microstructures were detected at the level of the DM and DM-stroma interface.

The posterior stroma seen from the *en face* view consisted of hyper-reflective stellate keratocytes along with linear structures against a hypo-reflective background (Figs 1f,i, 2e,h and 3d–g). Analogous to observations with confocal microscopy and electron microscopy, keratocytes in the µOCT images were stellate with one large body and several ramified processes (red asterisks in Figs 1f,i, 2e and h and 3d-g)^28, 29^. Those linear highly reflective structures could be classified into 3 categories on the basis of morphology, origin and travelling orientation: long cell processes of keratocytes, collagen fibres and nerve fibres. In mouse and rat stroma, fine collagen fibres predominantly ran in random orientations, thus making it difficult to differentiate them from thin nerve fibres (yellow arrows in Figs 1f,i, 2e,h, and Supplemental video 1); however, the nerve trunks (labelled ‘Y’ in Supplemental video 1) were easily detected, owing to their higher diameter compared with collagen fibres. By contrast, in swine stroma, comparably to second-harmonic generated (SHG) images^30^, collagen fibres tended to extend in parallel collagen bundles, interweaving with one another at specific orientations, and the density of visible collagen bundles gradually increased as they reached the DM (yellow arrows in Fig. 3d–f, Supplemental videos 2 and 3). Occasionally, high scattering cable-like structures with larger diameters than those of the collagen fibres ramified into branches, forming “Y”-shaped (labelled ‘Y’ in Supplemental videos 2 and 3) or asterisk-shaped (labelled * in Supplemental videos 2 and 3) structures in swine stroma; these structures were probably nerve fibres (yellow triangle in Fig. 3f and g and black arrows in Supplemental video 2).

### Longitudinal observation of endothelial decompensation

**Figure 4:**
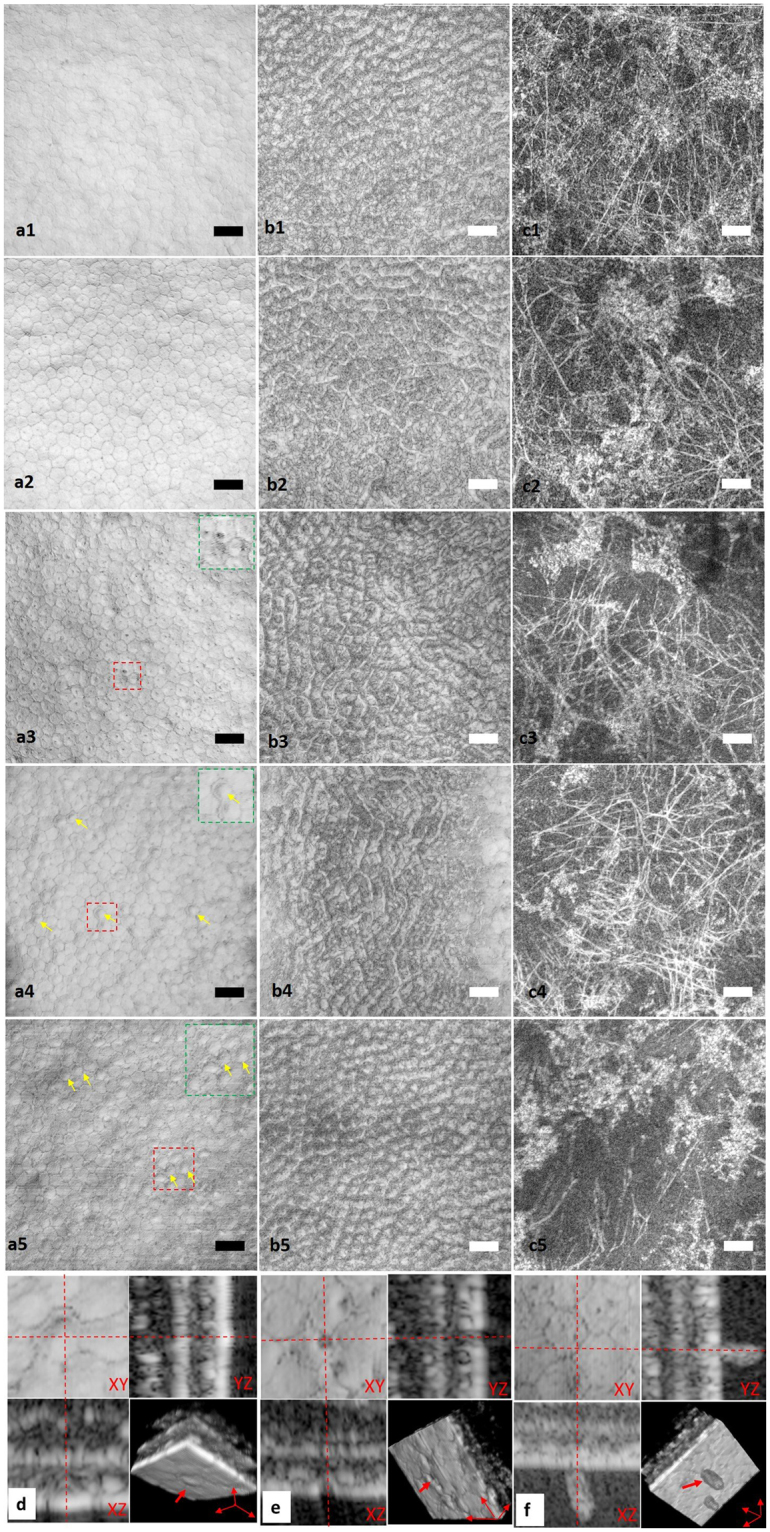
Longitudinal *ex vivo* observation of rat central cornea with µOCT. *En face* views taken at <5 mins (**a1**–**c1**), 2 h (**a2**–**c2**), 4 h (**a3**–**c3**), 8 h (**a4**–**c4**) and 24 h (**a5**–**c5**). (**a1**–**a5**) *En face* view of apical side of endothelial cells. Green square insets are zoomed-in view of red square areas in (**a3**,**a4** and **a5**), respectively. Yellow arrows in (**a4** and **a5**) indicate endothelial cell swelling, which changed the cell surface from flat to dome-shaped. (**b1**–**b5**) *En face* view of the basolateral surface of endothelial cells and the interface between endothelium and Descemet’s membrane. A high scattering lattice was detectable at all these time points. (**c1**–**c5**) *En face* view of posterior stroma. Highly reflective collagen fibres and keratocytes are both visible at different time points, whereas the density of collagen fibres appears to decrease at 24 hours after sacrifice. (**d**) 3D view of deformation of endothelial cells. Red arrow indicates dome-shaped deformation of endothelial cells at the apical surface. (**e**) 3D view of endothelial defect (red arrows). (**f**) 3D view of leukocyte infiltration into the cornea from cell boundaries (red arrow) (Scale bar = 50 µm).

To determine whether µOCT is effective for monitoring endothelial cell status after enucleation, µOCT images of the central cornea of the same rat were acquired at <5 mins and at 2, 4, 8, and 24 hours after harvest. Similarly to images acquired within 5 mins, the *en face* view of the posterior cornea captured at 2 hours demonstrated regularly arranged polygonal endothelial cells with dark spots at the apical surface, a lattice structure at the basolateral surface, and high scattering collagens and keratocytes in the stroma (Fig. 4a2–c2). After 4 hours, the area of the dark spots significantly increased, presenting an early sign of endothelial defect (rectangular area in Fig. 4a3). The lattices at the basolateral surfaces of endothelial cells as well as collagen fibres and keratocytes in the posterior stroma were all visible (Fig. 4a3–c3). After 8 hours, the plane of the endothelial apical surface was no longer as flat as it was at <5 mins. Several pimple-like or dome-shaped endothelial cells scattered amongst the flat hexagonal endothelial cells in cross-sectional images, thus indicating swelling of endothelial cell and providing evidence of endothelial decompensation (yellow arrows in Fig. 4a4,d). The rest of the components, including collagen and keratocytes, in the posterior cornea were well preserved (Fig. 4a4–c4). After 24 hours, it was difficult to differentiate cell boundaries, owing to the clustered swelling of endothelial cells leading to increased variation in endothelial cell morphology (rectangle area and yellow arrows in Fig. 4a5). Some endothelial cell defects were also observed at 24 hours, such as a gap in the continuous apical surface in the cross-sectional µOCT images (Fig. 4e). In addition, the density of collagen fibres was clearly decreased, thus indicating that pathological changes occurred in the stroma (Fig. 4c5). Several leukocytes in the anterior chamber were adhered to the endothelium, and the attachment point was located right at the cell boundary. This phenomenon indicated that leukocytes infiltrated the cornea via the interface between endothelial cells (red arrow, Fig. 4f).

In addition to morphological observation, total central corneal thickness (CCT), which is an important parameter in evaluation of endothelial function, was manually measured. The CCTs of rat cornea at <5 mins and at 2, 4, 8 and 24 hours were 189.12 ± 1.83 µm, 223.41 ± 0.84 µm, 250.01 ± 1.01 µm, 281.22 ± 1.34 µm and 354.72 ± 1.08 µm, respectively. One-way ANOVA and post hoc multiple comparisons indicated a statistically significant increase in CCT as early as 2 hours after enucleation (p < 0.001).

## Discussion

This study clarified what information µOCT can provide in imaging the posterior cornea *ex vivo* and *in vivo*, a question that has not been fully answered by previous studies. First, we reproduced our previous observations of hexagonal endothelial cells in *ex vivo* rat cornea in an additional two species, mice and minipigs. The results demonstrated the applicability of µOCT to corneal imaging of other animals and humans without a need for excision and tissue processing, thus eliminating artefacts introduced with histological methods. Second, but more importantly, we observed the micro-anatomic characteristics of the posterior cornea that were shared among all three species *in situ* and which have not previously been reported. On the basis of the existing and new micro-anatomical information uncovered by µOCT, this study further explored the potential clinical utility of µOCT in the context of corneal transplantation, because its ability to provide both high and isotropic spatial resolution and high imaging speed makes µOCT a unique tool for this task. Last, we conducted *in vivo* µOCT imaging of swine corneas to confirm our *ex vivo* findings and to evaluate the feasibility and limitations of anterior segment µOCT imaging in humans.

µOCT cross-sectional images delineated posterior corneal layers, including the PDL (visible in swine cornea), DM and endothelium, with high clarity, and thus, µOCT performed similarly to the OCT system recently developed by Bizheva, which has a 0.95 µm axial resolution in the cornea^23^. Accurately locating posterior corneal layers allows for precise separation of the endothelium and DM as well as the PDL from adjacent stroma and has valuable significance in corneal graft preparation and posterior corneal surgeries, especially in DMEK, PDEK and DALK^2, 23^. Moreover, precise delineation of the endothelium and DM also allows for separate thickness measurements of these two layers and should contribute to the long-term study of the natural history of endothelial decompensation in diseases such as Fuch’s endothelial dystrophy, which is characterized by progressive endothelial cell degeneration and guttae formation on thickened DM^4, 7^. More interestingly, the µOCT cross-sectional images clearly show that endothelial cells have a dome-shaped basolateral side facing the DM and a flat apical side facing the aqueous humour, analogous to observations in *in vitro* cultured endothelial cells^31^. Comparison of our results with images captured with transmission electron microscopy suggests that the dome-shaped pattern may be partly due to the existence of an endothelial nucleus^6^. Although the shape of corneal endothelial cells has been thoroughly investigated by using histological methods, this is the first time that the detailed morphology of endothelial cell has been uncovered in intact corneal tissue under natural conditions both *ex vivo* and *in vivo*. This new natural corneal morphological feature may open up a new approach to efficiently evaluate endothelial cell status.

The µOCT *en face* images provided morphological information regarding endothelial cells with unprecedented detail. In addition to polygonal endothelial cells at the apical surface, a highly reflective lattice was observed at the basolateral side of the endothelial cells, which has not previously been reported with confocal imaging or FF-OCT^14, 17, 18, 29^. The inability to detect such a lattice with IVCM is probably due to its insufficient axial resolving power^14, 29^, whereas its invisibility in FF-OCT, which also performs optical *en face* sectioning but with a higher axial resolution (1~2 µm) than IVCM, may partly be a result of preclinical changes in tissues or anatomical differences among species^17, 18^. Although it has not previously been described, the network-like structure deserves further research to reveal its physiological function and clinical meaning and also enrich our knowledge of corneal anatomy. The current µOCT study presents images of regularly arranged polygonal endothelial cells with a similar quality but with a much larger field of view (872 µm × 872 µm) as compared with noncontact SM (~300 µm × 350 µm) and contact IVCM (~400 µm × 400 µm) which may contribute to a more accurate morphological analysis of patient’s endothelial cells^12–14, 32^. The *en face* images together with the cross-sectional view indicate µOCT’s superiority by providing morphological endothelial cell information in three dimensions. Thus, it may become an alternative for evaluation of eye bank corneas, especially for the assessment of endothelial cells, which is currently performed by conventional light microscopy, SM and IVCM^9, 33^. In this study, we also performed a preliminary study with rat eyes to determine whether µOCT could be used to monitor pathological processes in enucleated corneas. Images captured at different time points indicate that µOCT is able to detect several signs of endothelial cell degeneration at an early stage in three dimensions. This capability would directly aid in pre- and postoperative evaluation of corneal grafts used for corneal transplantation.

In addition to comprehensive interpretation of endothelial cells, µOCT also has the ability to detect cellular components in the stroma. In this study, we focused on the posterior stroma which is anatomically close to the endothelium and DM. Similarly to IVCM^14, 29^, µOCT also can be used to visualize keratocytes, and the visibility of keratocytes allows for cell density assessment, which may accordingly aid in diagnosis of diseases such as keratoconus. µOCT is superior to IVCM because it also has the capacity to detect collagen fibres in stroma, and the visibility of collagen may be attributed to the difference in refractive index between collagen (n = 1.41) and the corneal extracellular matrix (n = 1.35)^34^. Observation of collagen fibres is helpful for increasing our understanding of the relationship between collagen orientation and corneal shape and consequently providing insight into the pathogenesis of myopia and hyperopia. Moreover, collagen fibre visualization should also aid in longitudinal analysis of diseases such as corneal ectasia, which presents as disorganization of collagen fibres in stroma^35^.

The limitations of current µOCT technology for imaging corneas *in vivo* include short depth of focus of the system and motion artefacts in the reconstructed *en face* images. To achieve cellular level transverse resolution, the depth of focus (DOF) of the current µOCT systems is ~30–40 µm, thus making it difficult to keep the area of interest within the depth of focus, owing to the axial motion of eyes. The DOF issue may be resolved by depth of focus extension techniques^36–39^. However, it’s worth mentioning that the axial field of view, defined as the axial range of 6 dB intensity roll-off is much larger than the DOF (~115 µm and 32.6 µm in air respectively). As a result, in log scale images, landmark features of corneas can still be clearly visualized over the entire cornea though at a lower lateral resolution. In addition, insufficient image acquisition speed with an A-line rate at 60 kHz (60 frames/s) remains a barrier for *in vivo en face* image analysis. The quality of *en face* images of corneal endothelium reconstructed from the 3D dataset was significantly compromised by the eye motion, and thus, neither apical or basolateral cellular structures could be clearly identified. An effective motion tracking algorithm and improvement in µOCT image acquisition speed may be promising solutions for decreasing motion artifacts^40^.

## Conclusion

This study comprehensively explored the utility of current µOCT technology in providing micro-anatomical information about posterior corneas in normal small and large animals *ex vivo* and normal large animals *in vivo*. The results provide new knowledge regarding the morphological appearance of corneal endothelial cells, including the dome-shape of endothelial cells and the hyper-reflective lattice at the endothelium-DM interface. In addition, µOCT also allows for visualization of keratocytes, collagen fibres and nerve fibres in stroma. All these findings may provide new opportunities for improving our understanding of corneal anatomy and disease development. To demonstrate such an opportunity, we linked the 3D morphological changes in endothelial cells to endothelial decompensation. The current study indicates that µOCT may be a promising tool in corneal transplantation processes, from donor graft preservation to graft preparation and from preoperative evaluation to postoperative follow-up. Further development in establishing ultrahigh speed µOCT as well as improvement in DOF is essential to make it more suitable for future human *in vivo* imaging.

## Methods

### Micro-optical coherence tomography

The construction of the µOCT system used in this study was similar to the system reported previously^41^.Whereas, to achieve higher axial resolution, we replaced the fused fiber coupler with a free-space beam splitter (BS008 and PAFA-X-4-B, Thorlabs, New York) and the axial resolution was measured to be ~1.6 µm in air. The calibrated axial resolution in the cornea was approximately 1.16 µm with a standard corneal refractive index of 1.375^34^. The probing beam was focused on the specimen through a 20× objective (M Plan Apo NIR 20×, Mitutoyo, Japan) with an effective numerical aperture of ~0.125, thus resulting in a full width at half maximum (FWHM) transverse resolution of ~2.4 µm. The images were acquired by raster scanning of a light spot in the transverse (x-y) plane and frequency domain ranging along the depth (z) direction. The field of view was 0.872 mm × 0.872 mm × 0.8 mm (x × y × z). The maximum scan rate was 60 kHz, corresponding to an imaging speed of 60 frames per second, in which one single frame is a cross-sectional (x-z) image.

### Tissue preparation and image acquisition

Two C57/bl6 mice (female, 8–9 weeks old) and 4 Sprague Dawley rats (female, 12–14 weeks old) were used for *ex vivo* study, and 2 Sus Scrofa minipigs (female, 2–3 years old) were used for *in vivo* and *ex vivo* study. For *ex vivo* studies, eyes of laboratory animals were immediately enucleated after sacrifice and washed using buffered saline (PBS) (pH = 7.4, Gibco^®^ by Life Technologies). Subsequently, each enucleated eye was placed into a custom-designed container with the corneal side up and transferred onto the scanning stage of the µOCT system for image acquisition. All images of the central cornea were captured by one researcher. After image acquisition, eyes were fixed with neutral-buffered formalin (4% formaldehyde; Leica Biosystems Richmond Inc.) for histological analysis. In addition, to test the capability of µOCT to longitudinally monitor the status of endothelial cells in corneal grafts, enucleated rat eyes were stored in PBS at 4 °C, and image acquisition was repeated at <5 mins and at 2, 4, 8, and 24 hours after harvest. For *in vivo* studies, experimental animals were mounted on the scanning stage for image acquisition while they were under general isoflurane anaesthesia. The study was approved by the Institutional Animal Care and Use Committee of Nanyang Technological University, Singapore (ARF-SBS/NIE-A0312) and the Institutional Animal Care and Use Committee of PWG Genetics Pte Ltd., Singapore (PN16076). All included animals were treated according to the statement of the Association for Research in Vision and Ophthalmology regarding the Use of Animals in Ophthalmic and Visual Research.

### Statistical analysis

Endothelial cell density of normal mice, rat and swine was evaluated using fixed-frame analysis^13^. Average endothelial thickness and DM thickness of each species were quantified based on 100 randomly selected positions of each three dimensional dataset. To compare CCT at different time points after harvest (<5 mins, 2, 4, 8 and 24 hours), 32 images were selected from 1024 cross-sectional images for quantitative analysis. One-way analysis of variance (ANOVA) was used to compare corneal thickness among different groups, and Bonferroni post hoc tests were performed for multiple comparisons when statistical significance was recognized. All numerical values are presented as the mean ± standard deviation (SD), and p < 0.05 was considered to indicate a statistically significant difference. All statistical analysis was performed using SPSS software (IBM SPSS Statistics 23.0).

### Data availability

The datasets generated during and/or analysed during the current study are available from the corresponding author on reasonable request.

## Electronic supplementary material

Supplemental video 1

Supplemental video 2

Supplemental video 3
